# Optic neuropathies that mimic glaucoma

**Published:** 2023-01-30

**Authors:** Michelle Dinsdale, José Manuel Guajardo-Beroiza, Jibran Mohamed-Noriega, Neeru Amrita Vallabh

**Affiliations:** Ophthalmologist: St. Paul's Eye Unit, Liverpool University Hospital NHS Foundation Trust, Liverpool, UK.; Ophthalmologist: Ophthalmology Unit, Clínica Alemana, Universidad del Desarrollo, Santiago, Chile.; Ophthalmologist: Department of Ophthalmology, University Hospital and Faculty of Medicine, Autonomous University of Nuevo Leon (UANL), Mexico.; Ophthalmologist: St. Paul's Eye Unit, Liverpool University Hospital NHS Foundation Trust and Department of Eye and Vision Science, University of Liverpool, Liverpool, United Kingdom.

## Abstract

It can be difficult to tell the difference between a glaucomatous optic nerve and a glaucoma mimic. Here are some tips that well help you to avoid diagnosing glaucoma when there is something else going on.

Glaucoma is a neurodegenerative disease of the optic nerve (optic neuropathy) and is characterised by the progressive, irreversible loss of retinal ganglion cells resulting in irreversible visual impairment and eventual blindness. Glaucoma is the most common optic neuropathy, with a global prevalence of 3.5% in people above the age of 40 years.^1^ There are many other causes of optic neuropathy, and these can easily be mistaken for glaucoma. This article will consider various ways that clinicians can differentiate between these by means of history taking, examination, investigations, and observation.

## History taking

- **Age.** Glaucoma usually (but not exclusively) affects people over the age of 50. If your patient is younger, consider an alternative diagnosis.
- **Symptoms.** Open-angle glaucoma is a painless and asymptomatic condition, unlike optic neuritis (which causes pain on eye movement) or an arteritic ischaemic optic neuropathy, which results in headache and discomfort when chewing (jaw claudication).
- **Length of time.** Primary open-angle glaucoma is a gradually progressive condition, whereas optic neuritis and ischemic optic neuropathies have an acute onset. Compressive causes tend to be subacute.
- **Previous ocular history.** Ask if your patient has previously had any episodes of optic neuritis or ischaemic optic neuropathy. This may not have been previously diagnosed or explained to them, so ask about previous symptoms which may suggest these.
- **Past medical history.** A history of vascular risk factors, such as raised blood pressure or cholesterol, may suggest a potential diagnosis of non-arteritic anterior ischaemic optic neuropathy.
- **Nutrition, drug, and alcohol use.** Patients with a poor or unbalanced diet are at risk of nutritional optic neuropathies, and those known to have alcohol dependence are at high risk of vitamin B12 deficiency. Intentionally inhaling volatile solvents or drinking methanol causes toxic neuropathy.

## Examination

- **Visual acuity.** Central vision, which is what is tested using a Snellen or LogMAR chart, is usually preserved in glaucoma, unless the patient has very advanced disease. However, central vision is often reduced much earlier in other optic neuropathies.
- **Colour vision.** This is often preserved in glaucoma until a very advanced stage, while in other causes of optic neuropathy it is often affected at an early stage. If colour vision is reduced, but the nerve looks reasonably preserved, this should encourage you to consider a glaucoma mimic. Colour vision is usually assessed using Ishihara colour plates; however, if these are not readily available, another option is to use a smartphone and the free Eye Handbook app,^2^ which has a colour vision testing section.
- **Intraocular pressure (IOP).** Although IOP is not always raised in glaucoma, if the pressure is below 21 mmHg, it is worthwhile considering whether this could be a glaucoma mimic.
- **Gonioscopy.** This allows assessment of the angle in the anterior chamber and may help to confirm the diagnosis of narrow-angle or angle-closure glaucoma, in which the IOP spikes intermittently.
- **Optic nerve.** This is best assessed during a dilated slit lamp examination (assuming the angle is open on gonioscopy) using a higher magnification lens; for example, a 78D or 60D lens. A dilated assessment with a magnified lens allows careful and close inspection of the nerve.

**Figure 1:**
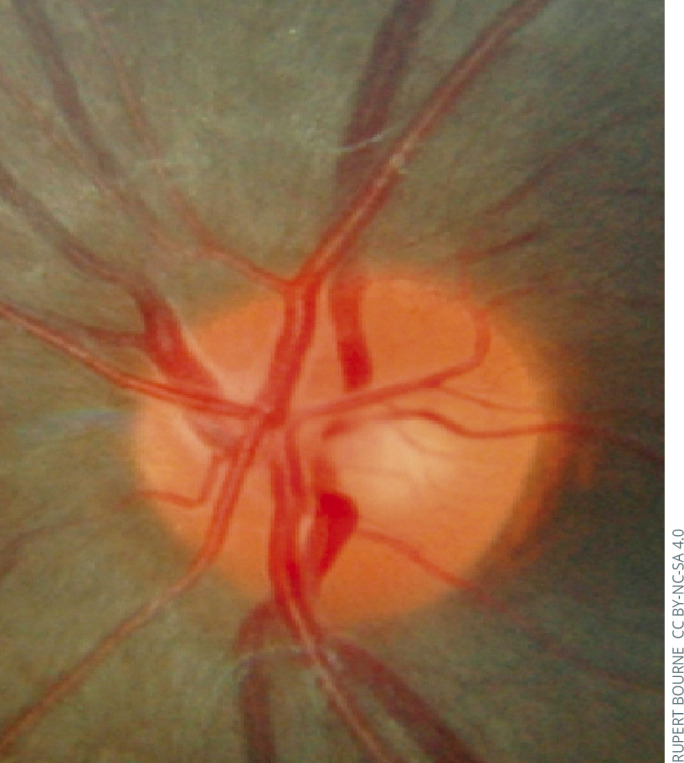
A healthy optic nerve. The rim is orange in colour with a small central cup which is yellow in appearance.^3^

## Examining the optic nerve

Look at the **vertical cup-to-disc ratio.** The central part of the nerve is called the cup and is yellow or white in colour, and the rim of the nerve is usually more orange in colour. The disc is the cup and the rim together, and the cup-to-disc ratio is the size of the cup, relative to the size of the whole disc. Figure 1 shows a healthy optic nerve.^3^

The orange rim becomes thinned in glaucoma, and thus the yellow cup becomes larger. **Enlargement of the cup-to-disc ratio** is one of the signs suggestive of glaucoma.

In glaucoma mimics, the cup-to-disc ratio is generally preserved so, generally, if the cup-to-disc ratio is less than 0.4, it is unlikely to be glaucomatous. A cup-to-disc ratio of greater than 0.8 is very suggestive of glaucoma, however. Between these ratios, you will need to rely on other findings to make a diagnosis.

**Figure 2:**
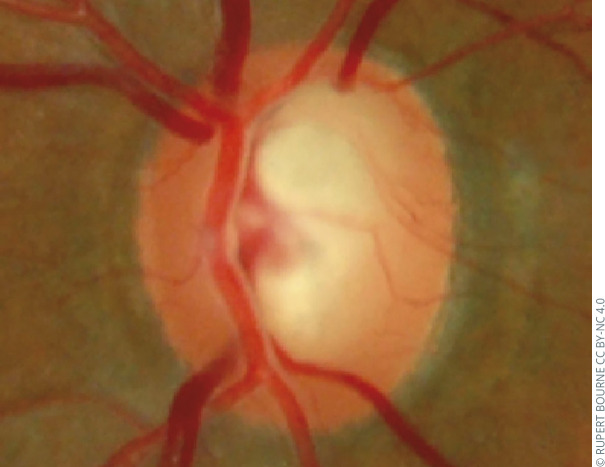
A cupped optic nerve. The centre of the optic nerve (the cup) is pale, but the rim remains a healthy colour; this is suggestive of glaucoma. Note that the cup-to-disc ratio in this image is greater than the cup-to-disc ratio in Figure 1.

The following are all suggestive of glaucoma:

- **A cupped optic nerve** (see Figure 2)^3^
- **Asymmetry of the cup-to-disc ratio** between the two eyes.
- **Optic disc haemorrhages** are often seen in unstable glaucoma^4^ and are not a feature of glaucoma mimics.^5^

The articles by Bourne and Khatib^3^ and Tsai^5^ offer useful guidance on assessing the optic nerve.

**Figure 3:**
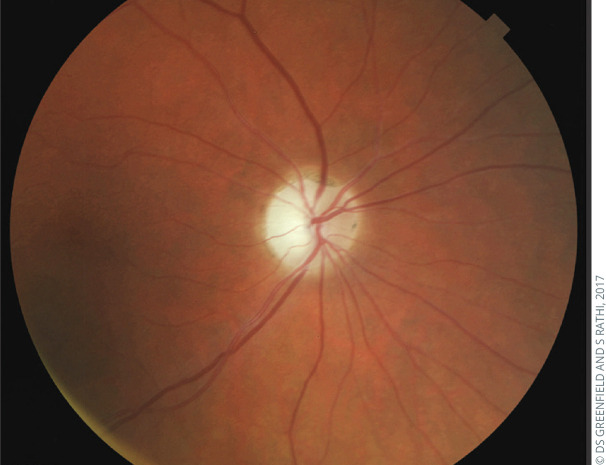
A colour photo of a pale optic nerve. The whole nerve is pale and this is described as disc pallor.^6^

It is important to look at the **colour of the nerve**. If, rather than having a healthy, orange-coloured rim, the whole nerve appears to be yellow/white in colour, this is described as optic disc pallor. Figure 3 illustrates a pale optic nerve/optic disc pallor.^6^ If you see **optic disc pallor**, consider another cause of optic neuropathy, i.e., a glaucoma mimic. You can still see optic disc pallor in glaucoma, but this is in end-stage glaucoma when the-cup-to disc ratio is more than 0.99.

Another warning sign for a unilateral optic neuropathy (or a glaucoma mimic) would be seeing **just one eye with optic disc pallor.**

Some optic neuropathies may initially present with **swelling of all or part of the optic nerve** prior to the onset of optic disc pallor. Glaucoma does not present as optic nerve swelling. Further techniques and training for optic nerve evaluation can be learnt using the online glaucomatous optic neuropathy evaluation (GONE) project.^7^

## Investigations: visual field assessment

Visual field assessment would ideally be performed using automated Humphrey visual field analysis or a Goldmann perimeter with an experienced operator. However, even visual fields to confrontation can give valuable information if performed well.^8^

Glaucoma affects the retinal nerve fibre layer (RNFL), which is anatomically distributed superiorly and inferiorly. Therefore, a glaucomatous visual field defect tends to affect the superior visual field or the inferior visual field and will progress within that hemifield prior to affecting the other hemifield.

**Figure 4:**
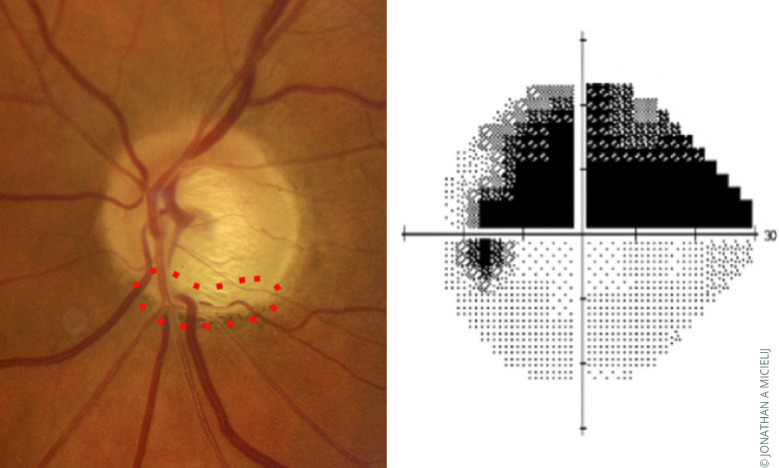
A superior visual field defect which does not cross the horizontal midline, highly suspicious of glaucoma. The red dotted circle indicates a corresponding thinning of the optic disc inferiorly.^9^

Figure 4 demonstrates a superior visual field defect^9^ which is sparing the inferior visual field; this is typical of glaucoma. **The visual field defect does not cross the horizontal midline** (referred to as respecting the horizontal midline). Glaucoma can also affect the inferior visual field and spare the superior visual field.

Furthermore, if the diagnosis is glaucoma, the visual field should correlate with the optic nerve assessment. For example, if the inferior rim of the optic nerve is thin, this will correlate with a superior visual field defect (as seen in Figure 4). If the optic nerve assessment and visual field do not correlate, you should consider whether this is a glaucoma mimic.

Due to the anatomy of the visual pathway posterior to the eye, a visual field defect which occurs due to a pathology behind the eye at either the chiasm or more posteriorly often affects the nasal or the temporal visual field.

**Figure 5:**
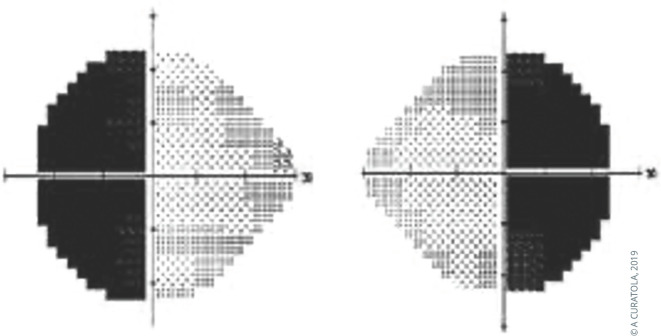
A visual field defect in the temporal aspect of both eyes which does not cross the vertical midline, highly suggestive of a non-glaucomatous lesion and suggests a lesion posterior to the eye, such as a neurological lesion.^10^

Figure 5 demonstrates a temporal visual field defect in both eyes where the nasal visual field is spared.^10^ This is **typical of a lesion behind the eye**, at the chiasm and therefore not typical of glaucoma. **The visual field does not cross the vertical midline** (referred to as respecting the vertical midline). If you see a visual field which respects the vertical midline, it is worth arranging some imaging of the brain, such as a coherence tomography (CT) or magnetic resonance imaging (MRI) scan.

### Observation of the patient

Glaucoma is a **progressive optic neuropathy** which means, the optic nerve appearance and function will deteriorate over time. Most (but not all) glaucoma mimics may be stable, such as a previous ischaemic optic neuropathy or a congenital disc anomaly. If the appearance is stable, it may be worth considering whether this is a glaucoma mimic.

## Alternative causes of an optic neuropathy

### Optic neuritis

This is due to inflammation of the optic nerve which can happen anywhere along its length. Typical optic neuritis is retrobulbar, so visual examination of the optic nerve may appear normal. It is typically caused by demyelination of the nerve and therefore may precede or be secondary to a diagnosis of a demyelinating disease.

### Ischaemic optic neuropathy

This occurs as a result of disruption of the blood supply to the optic nerve. There are two types:

- **Arteritic.** This is secondary to giant cell arteritis. In this condition, inflammation of the walls of the blood vessels causes narrowing of the blood vessels and thus reduces the blood supply to the optic nerve. This is an ophthalmic emergency requiring urgent systemic steroids. Patients have symptoms including a headache and discomfort or pain when chewing (jaw claudication).
- **Non-arteritic**. This affects patients with vascular risk factors. Atherosclerosis causes gradual narrowing of the blood vessels supplying the optic nerve. This eventually results in sudden optic nerve hypoperfusion and ischaemia.

### Compressive causes

Thisoccurs due to compression of the optic nerve or optic pathway, anywhere along its course. The signs and symptoms are therefore dictated by the location. Like other compressive causes, these are often progressive.

### Nutritional deficiencies, drugs, and alcohol

Nutritional optic neuropathy and toxic neuropathy are systemic causes of optic nerve damage and result in symmetrical symptoms – affecting both eyes equally. Patients with poor diets or nutrition are at risk of nutritional optic neuropathy, and vitamin B12 deficiency is particularly likely in those with alcohol dependence. A history of intentional inhalation of volatile solvents such as toluene, or consumption of methanol, can cause pale and cupped optic nerves with profound visual loss.

### Traumatic optic neuropathy

This can occur after trauma to the eye which is significant enough to damage the optic nerve.

The above is not an exhaustive list, but includes examples of some of the common causes of optic neuropathies, excluding glaucoma.

Remember, it is often difficult to differentiate between a glaucomatous optic nerve and a glaucoma mimic; even the best glaucoma experts struggle.^11^ However, applying the tips outlined in this article will help you to avoid diagnosing glaucoma when something else is going on.

**Signs that it may be a glaucoma mimic**

- Normal or low intraocular pressure
- Pale optic nerve, which is not cupped
- A visual field or OCT defect which respects the vertical midline but not the horizontal midline
- Reduced colour vision and a rapid afferent pupilalary defect (unless advanced)
- A stable/non-progressive optic neuropathy
- Young age
- Symptoms of headache or jaw claudication or pain
- Sudden onset

“It can be difficult to differentiate between a glaucomatous optic nerve and a glaucoma mimic; even the best glaucoma experts struggle.”

## Case study 1

**Figure 6:**
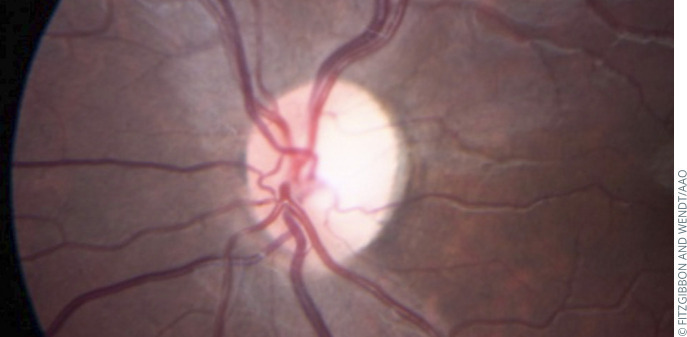
Left eye optic pallor.^12^

An 80-year-old woman was referred for a diagnosis of atypical glaucoma. Five years earlier she had a history of acute headache and visual loss in the left eye. At the time, she complained of stiffness around the shoulders and hips after resting, which was diagnosed as a rheumatological disease (polymyalgia rheumatica). Temporal artery biopsy also identified giant cell arteritis. Current ophthalmic examination revealed an asymmetric optic neuropathy with pallor and cupping only in the left eye (see Figure 6), probably due to a previous arteritic anterior ischaemic optic neuropathy.^12^

## Case study 2

**Figure 7:**
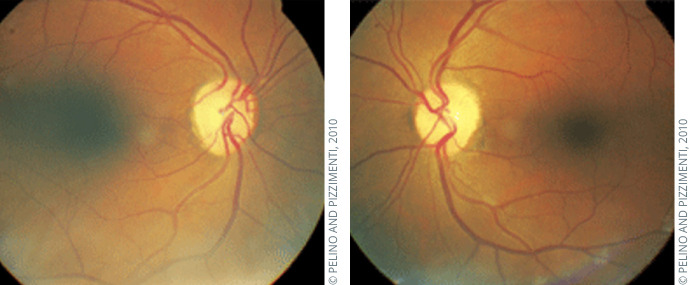
Image of bilateral optic nerve pallor.^13^

A 46-year-old patient presented at the eye clinic with a history of bumping into things over the last two months, and an awareness that her visual field was reduced. She also had a mild headache. Her visual acuity was normal, and her eye pressures were 12 mmHg in both eyes with open angles on gonioscopy. Her optic nerves demonstrated bilateral optic nerve pallor (Figure 7).^13^ She had a confrontational visual field test which demonstrated a visual field defect in the temporal aspect of both eyes (a bitemporal hemianopia) which respected the vertical midline. She went on to have a CT scan of her head, which demonstrated a pituitary lesion with compression of the optic nerve.
